# Pleomorphic liposarcoma of the extremity with solitary huge liver metastasis at initial diagnosis treated with conversion surgery combined with adjuvant chemotherapy: a case report

**DOI:** 10.1186/s13256-023-04223-2

**Published:** 2023-11-20

**Authors:** Seiji Shimomura, Toshiharu Shirai, Ryu Terauchi, Naoki Mizoshiri, Yuki Mori, Kanako Inuma, Shinji Tsuchida, Ryo Morimura, Hisashi Ikoma, Kenji Takahashi

**Affiliations:** 1 Department of Orthopaedics, Graduate School of Medical Science, Kyoto Prefectural University of Medicine, 465 Kajii-Cho, Kawaramachi-Hirokoji, Kamigyo-Ku, Kyoto, 602-8566 Japan; 2 Department of Digestive Surgery, Graduate School of Medical Science, Kyoto Prefectural University of Medicine, Kyoto, Japan

**Keywords:** Pleomorphic liposarcoma, Liver metastasis, Chemotherapy, Conversion surgery, Case report

## Abstract

**Background:**

Pleomorphic liposarcoma is the rarest subtype of liposarcoma. Pleomorphic liposarcomas are generally unresponsive to chemotherapy and radiotherapy. Moreover, metastasis in the liver, as the first and sole site, from a primary extremity soft tissue sarcoma, including pleomorphic liposarcoma, is extremely rare. Information regarding the appropriate management of these lesions is limited.

**Case presentation:**

A 50-year-old Japanese woman presented with a mass in the left thigh. Imaging examination revealed a soft tissue sarcoma on the left posterior thigh. The tumor was histologically diagnosed as pleomorphic liposarcoma. Computed tomography examination for assessment of metastases incidentally detected a huge liver mass. Wide excision of sarcoma was performed prior to chemotherapy. Right trisectionectomy was necessary to achieve hepatic clearance; however, the future liver remnant volume was insufficient. Therefore, we decided to administer anthracycline-based chemotheraphy to shrink the tumor. After seven courses of adriamycin-based chemotherapy, the liver tumor size was reduced from 211 mm × 106 mm × 180 mm to 105 mm × 66 mm × 90 mm. Finally, a right hemihepatectomy was performed. The patient was continuously monitored and was metastasis or local recurrence free within 5 months after liver surgery.

**Conclusion:**

Chemotherapy is effective in some cases for the treatment of unresectable liver metastases of pleomorphic liposarcoma, and complete resection is possible with conversion surgery. If the patient’s general condition permits, anthracycline-based chemotherapy can be used for the treatment of stage 4 pleomorphic liposarcoma.

## Background

The World Health Organization defines pleomorphic liposarcoma (PLPS) as a pleomorphic, high-grade sarcoma containing a variable number of pleomorphic lipoblasts. PLPS is the rarest subtype of liposarcoma, accounting for approximately 5% of all liposarcomas [1]. It commonly occurs in adults later in life, with a peak incidence in the seventh decade of life; its incidence is slightly higher in men than in women. PLPS is an aggressive sarcoma with local recurrence and metastatic rate of 30–50% and an overall survival (OS) rate of 60%. Metastases frequently occur in the lungs and pleura [2]. The liver as the first and sole site of metastases from a primary extremity soft tissue sarcoma is extremely rare [3]. Information regarding the appropriate management of these lesions is limited. We report the first case of PLPS in the left thigh with hepatic metastasis treated with chemotherapy and liver resection.

## Case presentation

**Fig. 1:**
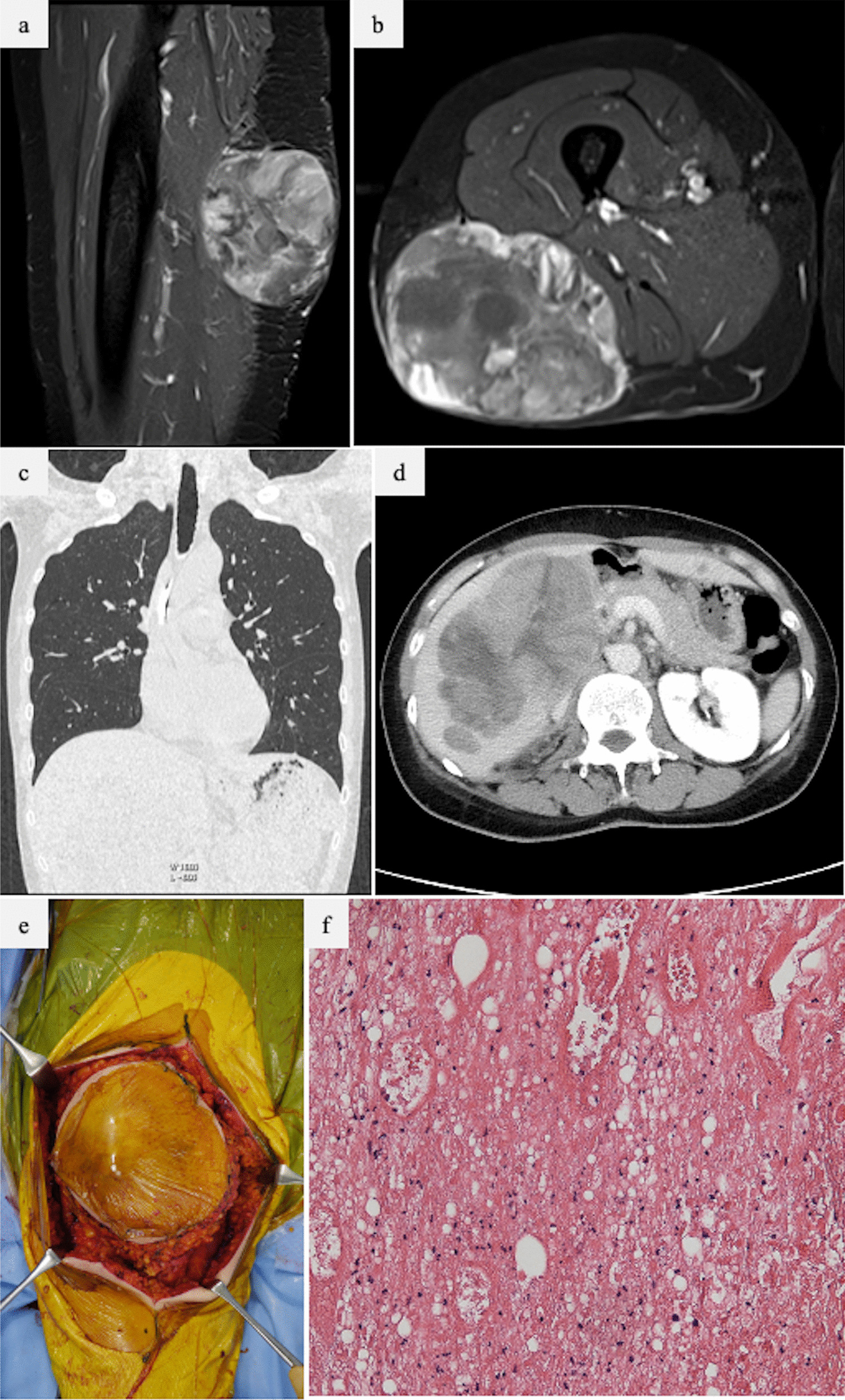
**a**, **b** MRI showing a tumor in the left thigh, with high signal intensity on T2-weighted images. **c**, **d** CT showing no remarkable lung metastasis (**c**), but huge metastasis in the liver (**d**). **e** Wide marginal excision of the left thigh. **f** View of the resected specimen, which was pathologically diagnosed as pleomorphic liposarcoma. *CT* computed tomography, *MRI* magnetic resonance imaging

We report the case of a 50-year-old Japanese woman who visited a local hospital owing to a palpable mass on the left thigh. The mass gradually increased in size and was associated with left thigh pain. The patient was referred to our hospital after 8 months. The patient had a history of hepatitis C virus (HCV) carrier, but no other major preexisting medical conditions were noted. Magnetic resonance imaging revealed the presence of sarcoma on the posterior left thigh (Fig. 1a, b), and differential diagnoses such as undifferentiated pleomorphic sarcoma, pleomorphic liposarcoma, dedifferentiated liposarcoma, and other higher-ranking sarcomas were considered. Then, needle biopsy confirmed the diagnosis of PLPS. During the performance of computed tomography (CT) examination to assess for metastases, a huge liver mass (140 mm × 80 mm × 120 mm) was found (Fig. 1c). A chest CT scan showed absence of lesions in the lungs or pleura (Fig. 1d). Needle biopsy of the liver mass revealed metastatic sarcoma of the PLPS in the left thigh. Liver function tests revealed normal bilirubin levels and mildly elevated transaminase levels. Meanwhile, the serum albumin level was low. Because the sarcoma on the thigh was large (113 × 91 × 94 mm) and nearly ulcerated on the skin, wide excision was performed prior to chemotherapy (CTx) (Fig. 1e). The tumor was resected from the biceps femoris muscle, and the excised tumor was submitted for pathological examination (Fig. 1f). A well-defined 15 × 12 × 10 cm mass was detected in the subcutaneous fatty tissue. A dense proliferation of round to short spindle-shaped cells of variable sizes, often with fatty droplets, was observed on histological examination. Some cells showed an adipoblast-like morphology. Large nuclei and multinucleated cells were also noted with prominent fission patterns and coagulative necrotic nests. Immunohistochemical staining showed that only a few of the tumor cells were positive for S-100 protein, while the majority were negative for cyclin-dependent kinase 4, murine double minute type 2, desmin, smooth muscle actin, cluster of differentiation 34, epithelial membrane antigen, and cytokeratin AE1/AE3; hence, a diagnosis of PLPS was made. Resection margins were negative (Fédération Nationale des Centres de Lutte Contre Le Cancer grade 3 (tumor differentiation: 3, tumor necrosis: 1, and mitotic score: 3).

**Fig. 2:**
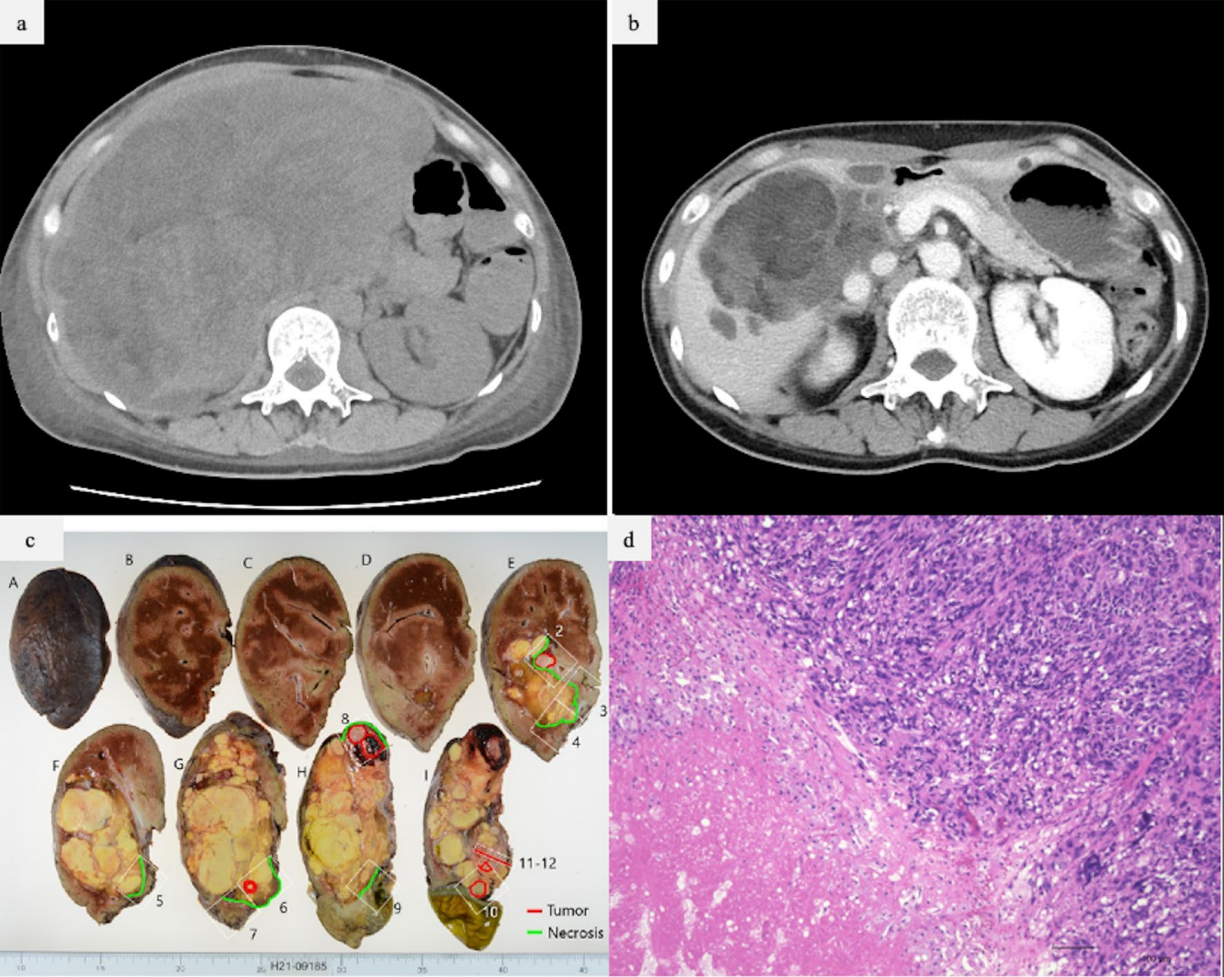
**a**, **b** CT showing the presence of metastatic liver tumor after surgery of the left thigh (**a**), and after seven courses of CTx (**b**). **c** Specimen obtained during the open right hemihepatectomy. **d** View of the resected specimen, which was pathologically diagnosed as a metastatic pleomorphic liposarcoma of the liver with 90% necrosis. *CT* computed tomography

At 4 weeks postoperatively, the metastatic liver tumor had increased to 211 × 106 × 180 mm in size (Fig. 2a), and symptoms of gastrointestinal obstruction developed. Chest CT scan showed absence of lesions in the lungs or pleura. Right trisectionectomy was necessary to achieve hepatic clearance. However, liver function tests revealed normal bilirubin levels, mildly elevated transaminase levels, and an indocyanine green retention rate at 15 min of 6%, but low serum albumin levels, huge tumor volume (669 ml), and insufficient future liver remnant (FLR; 23.7%). Therefore, we decided to administer anthracycline-based chemotherapy to shrink the tumor. The first course consisted of adriamycin and ifomide, but ifomide-induced encephalopathy occurred after the first dose; therefore, the second and subsequent courses were switched to adriamycin alone. After seven courses of adriamycin-based CTx, the liver tumor size was reduced to 105 mm × 66 mm × 90 mm, and the lesion was eventually confined to the right liver lobe (Fig. 2b). According to the Response Evaluation Criteria in Solid Tumours version 1.1, the target lesion showed partial response (PR) [211 mm → 105 mm (50% reduction)], and nontarget lesions and new lesions were not found; therefore, the overall outcome was judged to be PR. Preoperative percutaneous transhepatic portal vein embolization was performed to secure the FLR after the seventh course of CTx. Finally, a right hemihepatectomy was performed (Fig. 2c).

The excised liver weighed 782 g. Multiple masses measuring 20–45 mm in diameter protruded from the serous surface of the liver near the gallbladder. The masses were multinodular, fused, and measuring 110 × 100 × 55 mm, most of which appeared yellowish-white and necrotic. Histologically, most yellowish-white areas were composed of necrotic and calcified tissues and foam cell aggregates, with hemosiderin deposition and monocytic infiltration. The margins of some necrotic materials had small numbers of viable tumor cells. The tumor cells showed nuclear polymorphism and dark staining and contained cytoplasmic fatty droplets. The histological characteristics were similar to those of the tumor in the posterior aspect of the left thigh and consistent with those of a metastatic tumor. The margins were negative. The remaining proliferative tumor cells accounted for 10% of the original metastasis (Fig. 2d).

No major postoperative complications occurred, and the patient was discharged from the hospital on the ninth postoperative day. Metastasis or local recurrence did not occur.

## Discussion and conclusion

To the best of the authors’ knowledge, this case report is the first in which conversion surgery was performed as treatment for an unresectable metastatic liver tumor with soft tissue sarcoma as the primary tumor.

PLPS is a rare and aggressive subtype of liposarcoma. It typically occurs in the limbs and less commonly in the trunk or retroperitoneum. Distant metastases develop in 30–50% of patients, typically involving the lungs, and are unresponsive to chemotherapy or radiotherapy [4, 5]. Tumor-associated mortality occurs in 50% of patients [2].

The lungs are the most common metastatic site. Hepatic metastases from primary soft tissue sarcomas frequently present with visceral and retroperitoneal involvement [6]. Liver metastasis is uncommon in extremity or trunk soft tissue sarcomas (~ 0.5%) [6]. Tumor metastasis is rarely restricted to the liver. In a study of 637 patients with extremity or trunk soft tissue sarcomas, the liver was not the sole or first metastatic site [6]. Hasan *et al.* retrospectively identified metastatic disease in 630 patients with bone and soft tissue sarcomas and found that only 16 (12%) had metastasis to sites other than the lungs as a first or single occurrence. Of them, one patient developed liver metastasis from a chondrosarcoma, while liver metastasis did not occur in the only patient who had soft tissue sarcoma [3]. Conversely, 0.9% of liver tumors are classified as metastatic sarcomas, which rarely occur [7]. The histological type is a risk factor for visceral metastasis, including liver metastasis, and liposarcoma can metastasize to sites other than the lungs [8]. However, liposarcoma rarely metastasizes solely to the liver, as it frequently spreads to other body parts with the lungs as the most common metastatic site. Two patients with liver metastasis from PLPS have been reported in previous studies. One patient showed liver metastasis after primary tumor resection and chemotherapy [9], while the other patient was diagnosed with liver metastasis but had no treatment history or information on metastasis to other sites [10]. To date, no study has reported PLPS without pulmonary metastasis and with a first occurrence of liver metastasis. This is also an extremely rare case in which a patient with PLPS in extremity developed liver metastasis without lung metastasis on the first visit.

By contrast, metastasectomy for removal of metastatic soft tissue sarcoma in the liver prolongs the prognosis [8]. The presence of extrahepatic lesions and a tumor diameter of > 100 mm are poor prognostic factors in surgical cases of metastatic liver tumors from soft tissue sarcomas [11]. Moreover, the prognosis of patients with unresectable disease is extremely poor owing to the inadequate hepatic reserve, poor general condition, or metastases at other sites. Smolle *et al*. reported OS times of > 3 years in patients who were not potential candidates for metastasectomy, but less than 6 months in those who were inoperable and treated with chemotherapy [8]. Chemotherapy is a treatment option in many patients with inoperable disease, but its efficacy is often limited [12]. Some studies also reported the use of transcatheter arterial chemoembolization (TACE), while one study found that TACE was as effective as surgical resection for liver metastases from leiomyosarcoma [13]. In this case, chemotherapy and conversion surgery were effective in keeping the patient alive for more than 1 year after the initial diagnosis, and chemotherapy may be a treatment option for inoperable liver metastases.

In conclusion, we report herein an extremely rare case in which a patient with PLPS in extremity developed liver metastasis without lung metastasis at the first visit. Moreover, we report the first case of PLPS in extremity with hepatic metastasis treated with adjuvant chemotherapy and conversion surgery of liver resection. This case demonstrates the potential efficacy of chemotherapy and conversion surgery in treating unresectable metastatic liver tumors with primary soft tissue sarcoma.

## Data Availability

The datasets used and analyzed in the current study are available from the corresponding author upon reasonable request.

## Abbreviations

CT: Computed tomography
CTx: Chemotherapy
PLPS: Pleomorphic liposarcoma
